# Nucleoprotein of influenza A virus negatively impacts antiapoptotic protein API5 to enhance E2F1-dependent apoptosis and virus replication

**DOI:** 10.1038/cddis.2015.360

**Published:** 2015-12-17

**Authors:** A K Mayank, S Sharma, H Nailwal, S K Lal

**Affiliations:** 1 Virology Group, International Centre for Genetic Engineering & Biotechnology, Aruna Asaf Ali Road, New Delhi 110067, India; 2 School of Science, Monash University, Bandar Sunway, Petaling Jaya, Selangor DE 47500, Malaysia

## Abstract

Apoptosis of host cells profoundly influences virus propagation and dissemination, events that are integral to influenza A virus (IAV) pathogenesis. The trigger for activation of apoptosis is regulated by an intricate interplay between cellular and viral proteins, with a strong bearing on IAV replication. Though the knowledge of viral proteins and mechanisms employed by IAV to induce apoptosis has advanced considerably of late, we know relatively little about the repertoire of host factors targeted by viral proteins. Thus, identification of cellular proteins that are hijacked by the virus will help us not only to understand the molecular underpinnings of IAV-induced apoptosis, but also to design future antiviral therapies. Here we show that the nucleoprotein (NP) of IAV directly interacts with and suppresses the expression of API5, a host antiapoptotic protein that antagonizes E2F1-dependent apoptosis. siRNA-mediated depletion of API5, in NP-overexpressed as well as IAV-infected cells, leads to upregulation of apoptotic protease activating factor 1 (APAF1), a downstream modulator of E2F1-mediated apoptosis, and cleavage of caspases 9 and 3, although a reciprocal pattern of these events was observed on ectopic overexpression of API5. In concordance with these observations, annexin V and 7AAD staining assays exhibit downregulation of early and late apoptosis in IAV-infected or NP-transfected cells on overexpression of API5. Most significantly, while overexpression of API5 decreases viral titers, cellular NP protein as well as mRNA levels in IAV-infected A549 cells, silencing of API5 expression causes a steep rise in the same parameters. From the data reported in this manuscript, we propose a proapoptotic role for NP in IAV pathogenesis, whereby it suppresses expression of antiapoptotic factor API5, thus potentiating the E2F1-dependent apoptotic pathway and ensuring viral replication.

Apoptosis, the major form of programmed cell death, has been implicated in viral disease progression and pathogenesis of many viruses.^1^ Host cells employ the apoptotic pathway effectively to stall virus replication.^2^ Viruses on the other hand devise strategies to evade such host responses geared towards minimizing apoptosis in infected cells.^3^ Nevertheless, some pathogenic viruses such as influenza A virus (IAV) actively elicit apoptotic response upon infection, as a mechanism of cell death and virus propagation.^4, 5, 6, 7, 8, 9^ Thus, apoptosis plays a pivotal role in the influenza life cycle. Although in early stages of viral infection, IAV activates antiapoptotic signals through phosphatidylinositol 3-kinase/RAC serine/threonine protein kinase, heat-shock proteins^10, 11^ and JNK and NF-*κ*B signaling^12^ pathways to evade the host defense mechanism, in the later stages IAV triggers apoptosis through activation of intrinsic pathways in order to achieve enhanced viral replication and dissemination.^13, 14, 15^ IAV, an enveloped RNA virus of the family *Orthomyxoviridae*, possesses a genome of eight negative sense RNAs encapsulated by nucleoprotein (NP), encoding around 16 proteins.^16^ Given the limited coding capacity of IAV genome it is not surprising that IAV exploits host proteins extensively to its advantage.^17^ The exploitation of cellular apoptotic pathways has been exhibited by many proteins encoded by IAV, such as PB1F2, NS1, M1, M2, and lately NP, all targeting strategic points in the host apoptotic pathway.^15, 18, 19, 20, 21^ Of all the proteins encoded by IAV, NP is the most conserved and abundantly expressed. It is a multifunctional protein that plays important roles in apoptosis, transcription, replication, morphogenesis, and budding of the virus. Besides having crucial roles in the viral life cycle, NP also functions as a key adapter molecule mediating the cross talk between virus and host cell processes, owing to its ability to interact with a wide variety of both viral as well as cellular factors.^22^ In the present study, we show that NP of IAV induces apoptosis in host cells by repressing apoptosis inhibitor protein 5 (API5). API5 also known as antiapoptosis clone 11 protein, is 55 kDa apoptosis-inhibiting nuclear protein, first identified as a factor that prevented apoptosis in growth factor-deficient conditions.^23^ Besides being ubiquitously expressed in many human tissues, it is found to be upregulated in various cancers such as cervical cancer, prostate cancer, non-small cell lung cancer, and B-cell chronic lymphoid leukemia.^24, 25, 26, 27, 28^ Recently elucidated crystal structure of API5 revealed many protein–protein interaction modules such as HEAT and ARM repeats, suggesting API5 may interact with other cellular proteins.^29^ Mechanistically, API5 targets and interacts with acinus, a cellular protein involved in chromatin condensation and DNA fragmentation, to prevent acinus-mediated DNA fragmentation, and thus, apoptosis.^30^ Incidentally, API5 was identified in a genetic screen as a factor responsible for suppression of E2F1-dependent apoptosis, which was further shown to involve apoptotic protease activating factor 1 (APAF1)-mediated cleavage of initiator as well as effector caspases.^31^ In our study, we show that NP of different strains of IAV interacts specifically with API5 in IAV-infected mammalian cells. We examine the physiological effect of the NP–API5 interaction on cellular status of API5 and its role as a regulator of E2F1-dependent apoptosis. We show that NP quells the cellular levels of API5, thus promoting recruitment of E2F1 on *APAF1* promoter. Lastly, our study sheds light on the significance of this interaction on IAV-induced apoptosis and viral replication. Taken together, our study defines a novel antagonistic relationship between viral NP and host antiapoptotic protein API5, which impinges on IAV-induced apoptosis and viral propagation.

## Results

### NP of IAV interacts with human API5

A human lung cDNA library was screened using NP of IAV (A/chicken/Hatay/2004; H5N1) as bait, in a lexA-(Hybrid Hunter, Invitrogen, CA, USA) based yeast two-hybrid system.^32^ The screen led to the identification of API5 as an interactor of NP, confirmed using BLAST analysis (Supplementary Figure S1). The interaction between full-length API5 and NP was validated in yeast two-hybrid system, by cloning full-length human cDNA of API5 in frame with activation domain vector pYESTrp2, followed by co-transformation with pHybLex/Zeo-NP in L40 strain of yeast cells. The co-transformants tested positive for histidine prototrophy and *β*-galactosidase activity (Supplementary Figure S1), confirming that IAV NP interacted with full-length human API5 protein.

**Figure 1:**
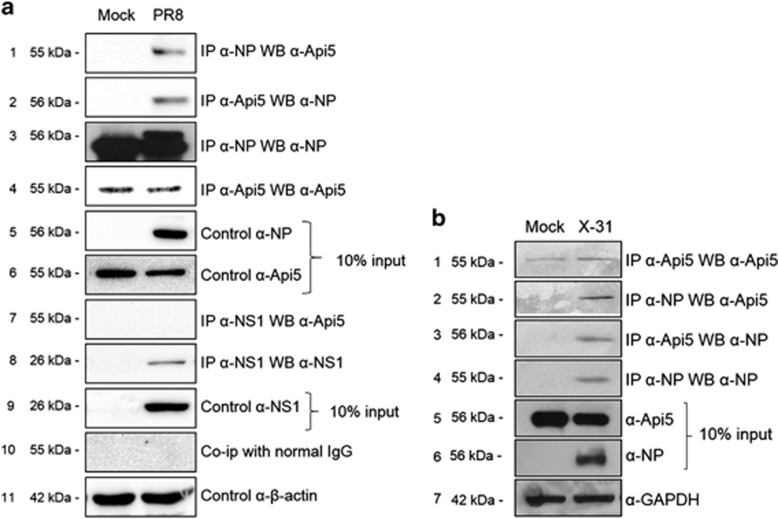
Influenza A virus nucleoprotein interacts with API5 in IAV-infected A549 cells. (**a**) Lung epithelial A549 cells were infected with PR8 at an MOI of 1. At 16 h post infection, cells were collected and lysed for co-immunoprecipitation assays. Panels 1 and 2 show immunoprecipitation of NP by *α*-NP followed by western blotting with *α*-API5 antibody and vice versa. Panels 3 and 4 show immunoprecipitation and western blotting with *α*-NP and *α*-API5 antibodies, respectively. Panels 5, 6, 9, and 11 show western blotting with *α*-NP, *α*-API5, *α*-NS1, and *α*-*β*-actin antibodies. Panels 7 and 8 show immunoprecipitation with *α*-NS1 (another IAV protein) followed by western blotting with *α*-API5 and *α-*NS1 antibody, respectively. Panel 10 shows the isotype control. (**b**) Lung epithelial A549 cells were infected with X31 at an MOI of 1. At 24 h post infection, cells were collected and lysed for co-immunoprecipitation assays. Panels 2 and 3 show immunoprecipitation of NP by *α*-NP followed by western blotting with *α*-API5 antibody and vice versa. Panels 1 and 4 show immunoprecipitation and western blotting with *α*-API5 and *α*-NP antibodies, respectively. Panels 5, 6, and 7 show western blotting with *α*-NP, *α*-API5, and *α*-GAPDH antibodies

The NP–API5 interaction was further ascertained by performing a co-immunoprecipitation (co-IP) assay in mammalian cells. To this end, A549 cells were infected with A/Puerto Rico/8/34 virus (H1N1; PR8) and A/Aichi/2/1968 (X31) at an multiplicity of infection (MOI) of 1. At 16 h post infection (p.i.), cells were collected and lysates were subjected to immunoprecipitation using antibodies specific for NP and API5. NP of PR8 and X31 virus co-precipitated with API5 (Figure 1a, panel 2 and Figure 1b, panel 3), and reciprocally API5 co-precipitated with NP (Figure 1a, panel 1 and Figure 1b, panel 2). Panel 3 of Figure 1a and panel 4 of Figure 1b show immunoprecipitation of NP followed by western blotting with anti-NP antibody, whereas Figure 1a panel 4 and Figure 1b panel 1 show immunoprecipitation of API5 followed by western blotting with anti-API5 antibody. As shown in panel 7 of Figure 1a, API5 does not interact with NS1, another influenza A viral protein, in PR8-infected cells, indicating the specificity of interaction between API5 and viral protein NP.

### NP of IAV colocalizes with API5 in the nucleus

**Figure 2:**
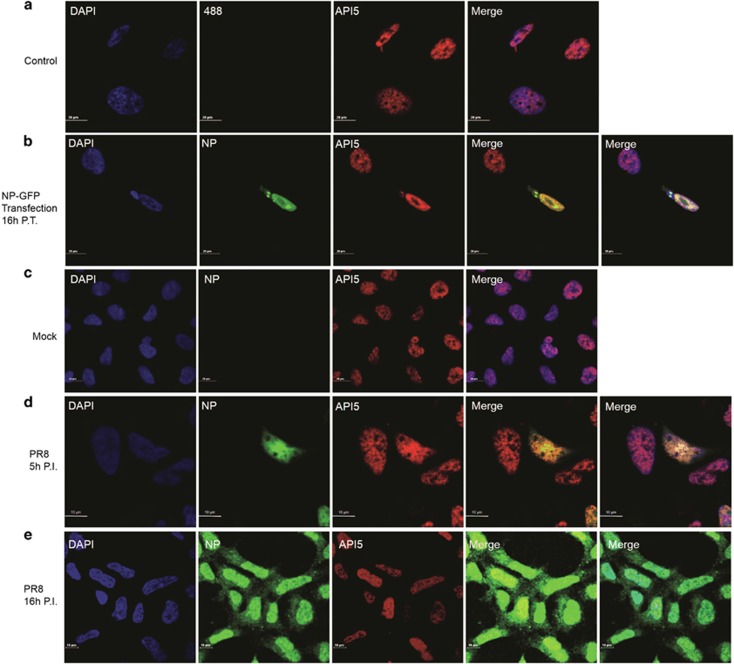
NP of IAV colocalizes with cellular API5. (**a** and **b**) A549 cells were transfected with either control (pcDNA3.1-myc/His) or NP-GFP (pEGFP-NP) plasmids. Cells were fixed with 2% paraformaldehyde 16 h post transfection (P.T.), followed by immunostaining using rabbit *α*-API5 primary antibody and Alexa594-conjugated anti-rabbit secondary antibody (red). NP is shown in green and nuclei are stained blue with DAPI. A549 cells were infected with either mock or PR8 at an MOI of 1 and fixed with 2% paraformaldehyde 5 h post infection (P.I.) (**d**) and 16 h post infection (**e**). API5 was stained using rabbit *α*-API5 as primary antibody and anti-rabbit conjugated with Alexa594 as secondary antibody (red). NP was stained with mouse *α*-NP as primary antibody and anti-mouse conjugated with Alexa488 as secondary antibody (green). Nuclei are stained blue with DAPI. (**a–c**) have a scale of 20 *μ*m and (**d** and **e**) have a scale of 10 *μ*m

Next, we sought to determine the intracellular site of interaction between NP and API5 in mammalian cells using two approaches. According to the first approach, A549 cells transfected with either control (pcDNA3.1-myc/His) or GFP-tagged NP (pEGFP-NP; Figures 2a and b) were fixed 16 h post transfection and subjected to immunofluorescence assay. In the second approach, A549 cells were infected with PR8 at an MOI of 1, and cells were fixed at 5 and 16 h p.i. followed by immunofluorescence (Figures 2c–e). As shown in the Figure 2b, NP of IAV colocalizes with API5 in the nucleus. Similar observations were also made in cells infected with PR8 strain of IAV (Figures 2d and e). It is interesting to note that API5 is a nuclear protein containing an NLS at its C terminus.^30^ Further, strong evidence suggests that API5 is a negative regulator of apoptosis induced by E2F1 (a nuclear protein).^31^ In light of these reports, nuclear colocalization of API5 with NP of IAV raises an interesting possibility that NP exploits E2F1-mediated apoptotic pathway via its interaction with API5.

### NP of IAV downregulates API5 expression

**Figure 3:**
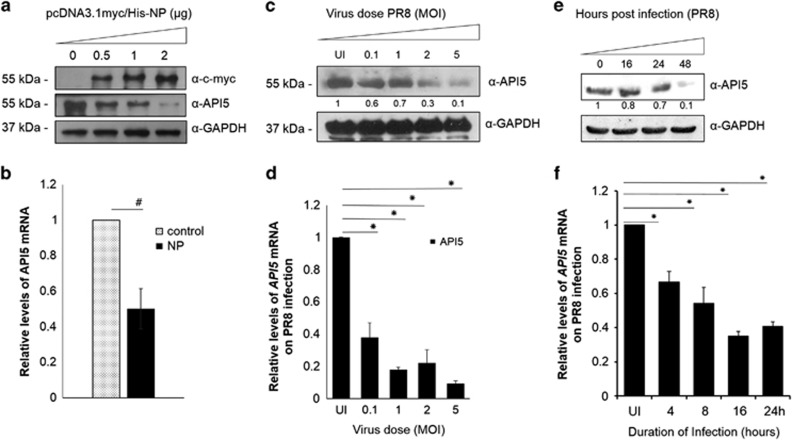
NP of IAV suppresses API5 expression. (**a**) A549 cells were transfected with either control (pcDNA3.1-myc/His) or with increasing concentration of myc-NP (pcDNA3.1-myc/His-NP), 48 h post transfection the whole-cell lysates were resolved on SDS-polyacrylamide gel electrophoresis (SDS-PAGE) for detection of NP, API5, and GAPDH using *α*-c-myc, *α*-API5, and *α*-GAPDH antibodies, respectively. (**b**) A549 cells were transfected with either control (pcDNA3.1-myc/His) or myc-NP (pcDNA3.1-myc/His-NP), 24 h post transfection the total RNA was isolated for the estimation of *API5* mRNA levels using quantitative PCR with specific primers. (**c**) A549 cells were either mock infected (MI) or infected with PR8 at an MOI of 0.1, 1, 2, and 5 for 24 h. The whole-cell lysates from the samples were resolved on SDS-PAGE for detection of NP, API5, and GAPDH. (**d**) A549 cells were either MI or infected with PR8 at an MOI of 0.1, 1, 2, and 5 for 24 h. Total RNA was isolated for the estimation of *API5* mRNA levels using quantitative PCR with specific primers. (**e**) A549 cells were infected with PR8 at an MOI of 1 and samples were collected at 0, 16, 24, and 48 h post infection (p.i.). The whole-cell lysates from the samples were resolved on SDS-PAGE for detection of API5 and GAPDH. (**f**) A549 cells were either MI or infected with PR8 at an MOI of 1 and samples were collected at 4, 8, 16, and 24 p.i. Total RNA was isolated for the estimation of *API5* mRNA levels by quantitative PCR using specific primers. Fold change in the expression levels of API5 protein are shown below each panel. The data in **b**, **d**, and **f** are shown as mean±S.D. of three independent experiments. # and * indicate statistically significant difference at *P*<0.05 and *P*<0.01, respectively

Next, we were interested in assessing the cellular status of API5 both in presence of NP and the whole virus. To this end, we examined the effect of transiently transfected NP on *API5* transcript, and increasing dosage of transiently transfected NP on API5 protein. As evident from Figures 3a and b, *API5* mRNA expression is suppressed by NP, and also API5 protein levels are negatively correlated to cellular NP levels when compared with control.

As a further test of impact of NP on API5 levels, we investigated the effect of virus dose and duration of infection on API5. As shown in Figures 3c and d, significant and progressive decrease in the levels of API5 mRNA and protein was observed upon infecting A549 cells with PR8 virus at increasing MOI when compared with uninfected A549 cells. Further substantiating our observations, a time-dependent decrease in relative levels of API5 mRNA and protein was recorded upon infecting A549 cells with PR8 (Figures 3e and f). Collectively, these results indicate a negative impact of IAV infection as well as NP on expression of API5. Thus, there is a high possibility that function of API5 as a suppressor of E2F1-dependent apoptosis may also suffer a setback under these conditions.

### Induction of APAF1-dependent apoptotic pathway by NP of IAV

**Figure 4:**
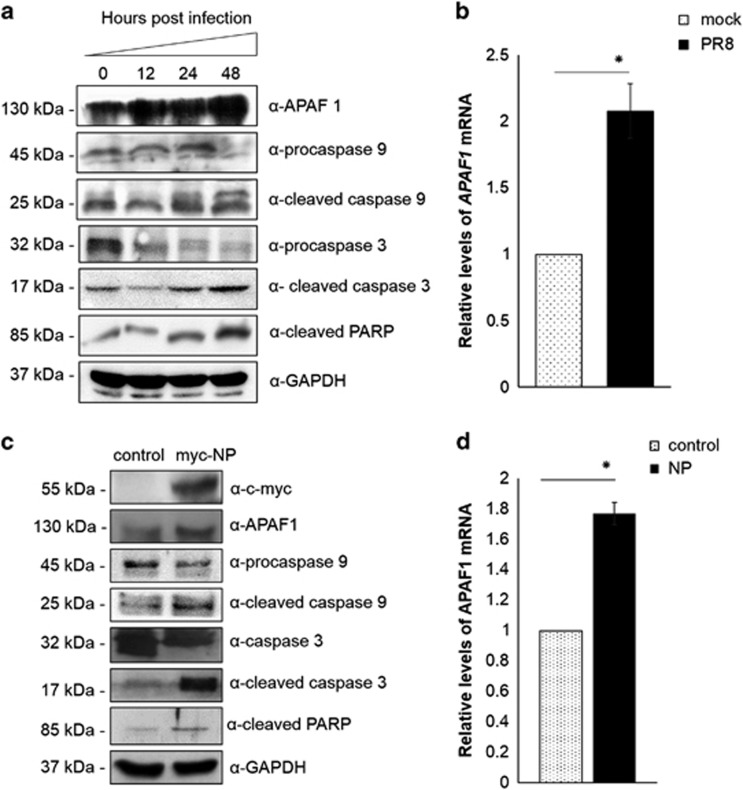
NP of IAV induces APAF1-dependent cleavage of initiator and effector caspases. (**a**) A549 cells were infected with PR8 at an MOI of 1. The whole-cell lysates were prepared at 0, 12, 24, and 48 h post infection, and were subjected to western blot analysis using indicated primary antibodies. (**b**) A549 cells were either mock infected (mock) or infected with PR8 (PR8) at an MOI of 1. At 24 h post infection, the total RNA was isolated for the estimation of *APAF1* mRNA levels by quantitative PCR using specific primers. (**c**) A549 cells were transfected with either control (pcDNA3.1-myc/His) or myc-NP (pcDNA3.1-myc/His-NP), and the whole-cell lysates were prepared at 48 h post transfection. The lysates were subjected to western blot analysis using indicated primary antibodies. (**d**) A549 cells were transfected with either control (pcDNA3.1-myc/His) or myc-NP (pcDNA3.1-myc/His-NP). At 48 h post transfection, the total RNA was isolated for the estimation of *APAF1* mRNA levels by quantitative PCR using specific primers. The data in **b** and **d** are shown as mean±S.D. of three independent experiments. *indicates statistically significant difference at *P*<0.01

Previous studies have shown that E2F1-mediated apoptosis involves APAF1-dependent induction of both initiator and effector caspases.^33^ In order to study the effect of NP–API5 association on E2F1-dependent apoptosis, we monitored time-dependent cellular status of APAF1 and initiator and effector caspases in IAV-infected A549 cells. Influenza virus is known to induce apoptosis during later stages of infection in order to facilitate effective viral propagation and dissemination.^34^ Concurrent with these findings, we observed a time-dependent increase in APAF1 protein levels, cleaved forms of both caspases 9 and 3 in infected cells (Figure 4a), indicative of progressive apoptosis. PARP cleavage was used as a marker of caspase activity. In addition, the relative levels of *APAF1* mRNA were elevated significantly (~2-fold) in infected cells (Figure 4b). Interestingly, overexpression of NP in A549 cells mirrored the effect of IAV infection on cellular apoptosis. As observed during IAV infection, NP overexpression (pcDNA3.1-myc/His-NP) too was marked by elevated levels of cleaved caspases and PARP, underscoring role of NP in IAV-induced apoptosis (Figure 4c). Importantly, *APAF1* mRNA levels were also increased (Figure 4d) indicating involvement of E2F1-dependent apoptosis in IAV infection via cross talk with API5.

### API5 inhibits apoptosis in infected cells

**Figure 5:**
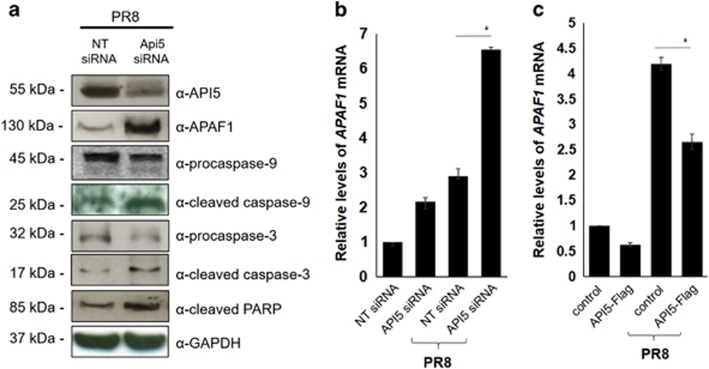
API5 promotes antiapoptotic effect in infected cells. A549 cells were transfected with either non-targeting (NT siRNA) or API5-specific siRNA (API5 siRNA). At 24 h post transfection, cells were infected with PR8 at an MOI of 1. At 24 h post infection, (**a**) the whole-cell lysate was resolved on SDS-polyacrylamide gel electrophoresis for detection of API5, APAF1, PARP, caspases 9 and 3 using indicated primary antibodies. (**b**) A549 cells were transfected with either NT siRNA or API5 siRNA. At 24 h post transfection, cells were infected with either mock or PR8 at an MOI of 1. At 24 h post infection, the total RNA was isolated for the estimation of *APAF1* mRNA by quantitative PCR using specific primers. (**c**) A549 cells were transfected with either control (pLPC) or API5-Flag (pLPC-API5-Flag), 24 h post transfection cells were infected with either mock or PR8 at an MOI of 1. At 24 h post infection, the total RNA was isolated for the estimation of *APAF1* mRNA by quantitative PCR using specific primers. The data in **b** and **c** are shown as mean±S.D. of three independent experiments. * indicates statistically significant difference at *P*<0.01

Our observations that IAV stimulates E2F1-dependent apoptosis and that it also directs transcriptional downregulation of antiapoptotic factor API5, prompted us to assess the impact of altering cellular status of API5 on IAV-induced apoptotic signaling. We thus analyzed the effect of siRNA-mediated knockdown of API5 on E2F1-mediated apoptosis. To this end, A549 cells depleted of API5 were infected with PR8 at an MOI of 1. As depicted in Figure 5a, there is a significant decrease in the levels of procaspases 3 and 9, and concomitant increase in the levels of their cleaved products along with increased PARP cleavage in infected cells treated with API5-specific siRNA when compared with scrambled siRNA. Moreover, relative levels of *APAF1* mRNA as well as protein increased markedly upon knockdown of API5 in infected cells (Figures 5a and b). On the contrary, API5 overexpression in infected cells led to the transcriptional suppression of *APAF1* (Figure 5c). These observations strongly indicate a negative regulatory role of API5 in IAV-induced apoptosis, which is countered by NP-mediated repression of API5.

### API5 impairs recruitment of E2F1 on *APAF1* promoter in infected cells

**Figure 6:**
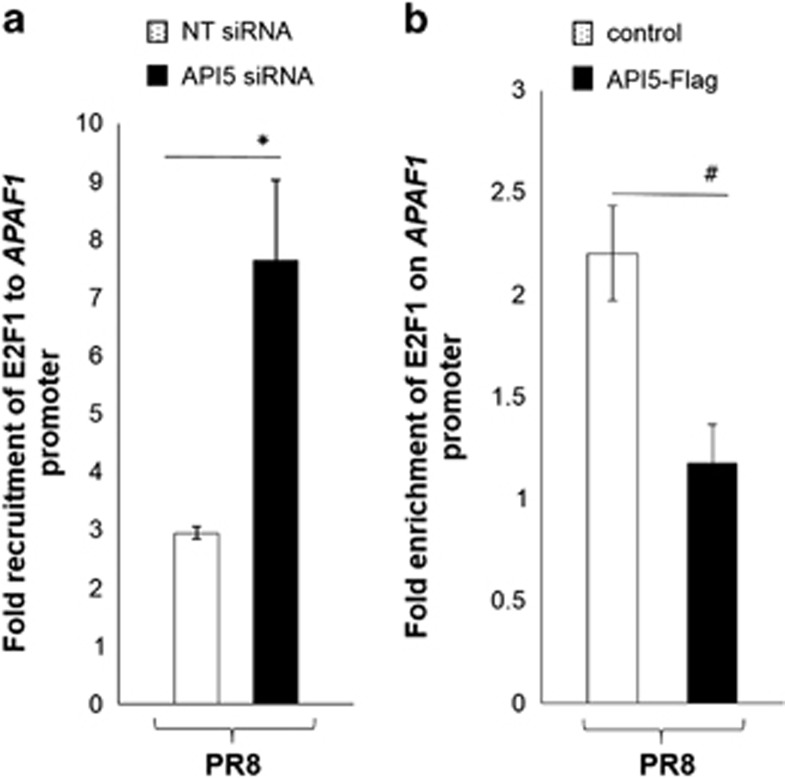
API5 alters E2F1 recruitment on *APAF1* promoter. A549 cells were transfected with (**a**) either non-targeting siRNA (NT siRNA) or API5-specific siRNA (API5 siRNA), (**b**) either control (pLPC) or API5-Flag (pLPC-API5-Flag). At 24 h post transfection, cells were infected with PR8 at an MOI of 1. Cells were fixed 24 h post infection for isolation of protein–DNA complexes and immunoprecipitated with E2F1 antibody. The genomic DNA bound to E2F1 antibody was isolated and analyzed by quantitative PCR using *APAF1* primers. The data are shown as mean±S.D. of three independent experiments. # and * indicate statistically significant difference at *P*<0.05 and *P*<0.01, respectively

It is well established that E2F1-dependent apoptosis involves transcriptional activation of target genes such as *APAF1* by E2F1 transcription factor, which further entails physical binding of E2F1 to target gene promoters.^35^ In order to delineate mechanistic details of antiapoptotic effects of API5 on E2F1-triggered apoptotic response in IAV-infected cells, we performed ChIP assay to investigate the status of E2F1 recruitment to *APAF1* promoter under conditions of API5 depletion and overexpression in infected cells. As shown in Figure 6a, siRNA-mediated knockdown of API5 expression facilitated increased recruitment of E2F1 to *APAF1* promoter. By contrast, enforced expression of API5 in infected cells led to abrogation of E2F1 occupancy on *APAF1* promoter (Figure 6b). On the basis of these observations, we conclude that API5, in order to obstruct the proapoptotic pathway, interferes with recruitment of E2F1 transcription factor to target gene promoters such as *APAF1*. Hence, it is of paramount importance for IAV to direct downregulation of this antiapoptotic factor, in order to ensure successful viral infection and spread.

### API5 overexpression suppresses apoptosis in IAV-infected as well as NP-transfected cells

**Figure 7:**
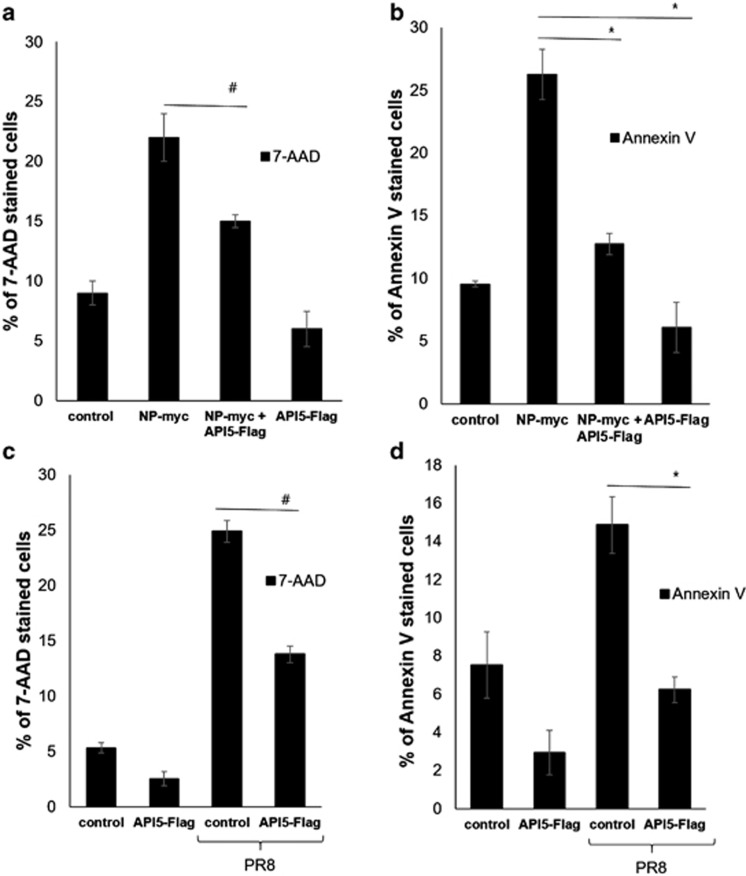
API5 suppresses NP-induced apoptosis. (**a** and **b**) A549 cells were transfected with either control (pcDNA3.1-myc/His), NP-myc (pcDNA3.1-myc/His-NP), API5-Flag (pLPC-API5-Flag) or co-transfected with NP-myc and API5-Flag. At 48 h post transfection, cells were collected and stained with either FITC-conjugated annexin V or 7AAD and subjected to flow cytometry, and were later analyzed using BD Cell Quest pro software (NJ, USA) and plotted as graphs. (**c** and **d**) A549 cells were transfected with either control (pcDNA3.1-myc/His) or API5-Flag (pLPC-API5-Flag). At 24 h post transfection, cells were infected with PR8 at an MOI of 1. Cells were collected 24 h post infection and stained with FITC-conjugated annexin V or 7AAD and subjected to flow cytometry, and were later analyzed using BD Cell Quest and plotted as graphs. For acquisition 20 000 cells per sample were used. The data are shown as mean±S.D. of three independent experiments. # and * indicate statistically significant difference at *P*<0.05 and *P*<0.01, respectively

Having assessed the effect of API5 on E2F1-dependent transactivation of proapoptotic genes under conditions of infection, we next set out to investigate its impact on the process of apoptosis itself. First, cells were transfected with either control, NP (NP-myc) alone, API5 (API5-Flag) alone or both constructs. Second, cells transfected with either control or API5 (API5-Flag) expression construct followed by infection with PR8 strain. In both cases cells were subjected to analysis for apoptosis using annexin V (an early apoptotic marker) and 7AAD (a late apoptotic marker) staining. As evident in Figures 7a and b, NP of IAV possesses a proapoptotic function, which is in agreement with previous reports.^21^ NP induces ~2-fold increase in 7AAD positive and ~2.5-fold increase in annexin V positive cells, when compared with control. Interestingly, API5 overexpression mitigates the proapoptotic effect of NP and significantly decreases the percentage of annexin V and 7AAD stained cells. Further substantiating these observations, cells stained positive for annexin V and 7AAD under conditions of IAV infection decreased markedly upon overexpression of API5 (Figures 7c and d). In all, these observations clearly demonstrate that NP and IAV induce apoptosis in cells, which is significantly attenuated by overexpression of API5.

### API5 suppresses viral replication

**Figure 8:**
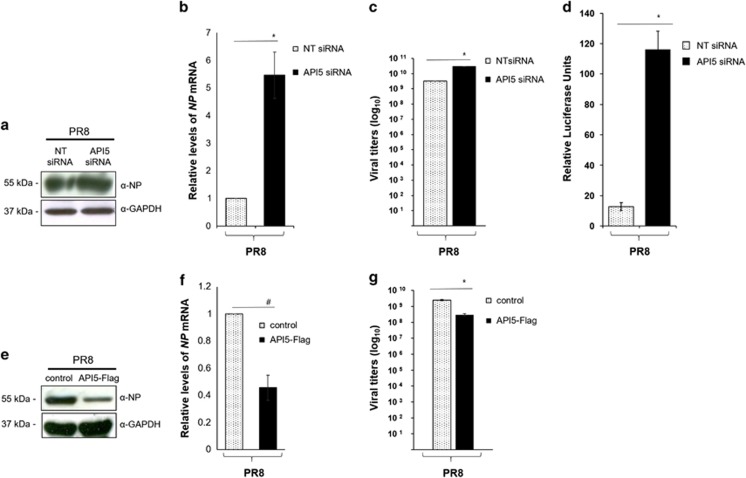
Effect of cellular status of API5 on viral replication. A549 cells were transfected with either non-targeting siRNA (NT siRNA) or API5-specific siRNA (API5 siRNA) for 24 h followed by infection with PR8 virus at an MOI 1. The cells were collected at 24 h post infection. (**a**) The whole-cell lysate was resolved on SDS-polyacrylamide gel electrophoresis (SDS-PAGE) and western blotted with *α*-NP and *α*-GAPDH antibodies, respectively. (**b**) Total RNA was isolated for the estimation of *NP* mRNA levels using quantitative PCR with specific primers. (**c**) A549 cells were transfected with either NT siRNA or API5 siRNA for 24 h followed by infection with X31 virus at an MOI 1. The cells were collected at 24 h post infection. Aliquot of supernatant obtained from IAV-infected cells was used to infect MDCK cells followed by the determination of viral titers using a plaque assay. (**d**) Plasmids encoding polymerase complex components (PA, PB1, PB2, and NP) derived from PR8 (H1N1 virus) were co-transfected alongside a reporter plasmid containing noncoding sequence from the NS1 segment of influenza A virus and luciferase gene driven by the pol I promoter in A549 cells that had been pretreated with either NT or API5 siRNA. Plasmid pRL-TK (Promega, WI, USA), which expresses renilla luciferase, was co-transfected as an internal control for data normalization. (**e**) A549 cells were transfected with either control (pcDNA3.1-myc/His) or API5-Flag (pLPC-API5-Flag) for 24 h followed by infection with either mock (allantoic fluid) or PR8 at an MOI 1. The cells were collected at 24 h post infection. The whole-cell lysate was resolved on SDS-PAGE and western blotted with anti-NP and anti-GAPDH antibodies, respectively. (**f**) Total RNA was isolated for the estimation of *NP* mRNA levels using quantitative PCR with specific primers. (**g**) A549 cells were transfected with either control (pcDNA3.1-myc/His) or API5-Flag (pLPC-API5-Flag) for 24 h followed by infection with X31 virus at an MOI 1. The cells were collected at 24 h post infection. Aliquot of supernatant obtained from IAV-infected cells was used to infect MDCK cells followed by the determination of viral titers using a plaque assay. The data in **b**, **c**, **d**, **f** and **g** are shown as mean±S.D. of three independent experiments. # and * indicate statistically significant difference at *P*<0.05 and *P*<0.01, respectively

Our findings thus far have highlighted an inhibitory role of API5 in IAV-infected cells, which is exercised via regulation of the E2F1–APAF1 apoptotic pathway. Further, our results also indicate that suppression of API5 by NP is instrumental in ensuring IAV-induced apoptosis. In this regard it is worthy to note that IAV-mediated apoptosis of infected cells favors viral replication and propagation.^36, 37^ Considering that API5 plays a critical role in IAV-induced apoptosis, it was imperative for us to evaluate the significance of API5 in IAV replication. For this purpose, we quantified the levels of *NP* mRNA in IAV-infected cells under conditions of API5 depletion and overexpression. Upon knockdown of API5 expression in A549-infected cells there was a significant upregulation of relative mRNA levels of *NP* when compared with control (Figure 8b). Conversely, enforced expression of API5 led to the downregulation of relative mRNA levels of *NP* (Figure 8f). In addition, NP protein levels were in agreement with mRNA levels under conditions of API5 depletion and overexpression in infected cells (Figures 8a and e). Importantly, API5 depletion in infected cells led to ~5-fold increase in the viral titers as determined by plaque assay when compared with control (Figure 8c). Conversely, API5 overexpression severely attenuated viral titers (~6-folds) (Figure 8g). These results were further confirmed using a replicase assay, wherein A549 cells were treated with either non-targeting siRNA or API5-specific siRNA followed by co-transfection with plasmids encoding PR8 polymerase complex genes PB2, PB1, PA, and NP in conjunction with a reporter plasmid containing the UTR of the NS1 segment upstream of the luciferase gene driven by the human RNA pol I promoter. Strengthening our previous observations, replicase assay results showed sixfold elevation in replicase activity upon depletion of API5 (Figure 8d). Upregulation of *NP* mRNA, protein levels, polymerase activity as well as viral titers in API5-depleted cells are clear indicators that viral replication is inhibited by the host factor API5. NP interacts and downregulates API5 to relieve suppression of the E2F1–APAF1 apoptotic pathway.

Taken together, our findings argue in favor of a critical role of antiapoptotic protein API5 in IAV-induced apoptosis and virus replication. NP–API5 association has a negative bearing on cellular levels of API5, which mitigates API5-induced suppression of E2F1–APAF1-dependent apoptotic signaling thus favoring virus propagation.

## Discussion

Apoptosis is an integral part of pathogenesis of many viruses such as human immunodeficiency virus type 1, chicken anemia virus, feline leukemia virus, and papillomavirus.^38, 39, 40, 41^ Likewise, in IAV infection apoptosis is a major contributor of cell death and tissue damage.^42^ It is still debatable whether apoptosis favors virus or host, but recent studies give evidence that IAV inhibits apoptosis by activating anti-apoptotic signals during early stages. However in the later stage of the IAV life cycle, it disables antiapoptotic pathways for efficient viral replication and dissemination. IAV proteins such as PB1F2, HA, M1, M2, and recently NP have been demonstrated to participate in IAV-induced apoptosis.^15, 18, 19, 20, 21^ The virus does so by hijacking various pathways and functions of host-cellular proteins. Several cellular signaling pathways hijacked by the virus have been studied, including the phosphatidylinositol 3-kinase (PI3 K)/Akt pathway, MAPKinase pathway or the protein kinase C (PKC)/protein kinase R (PKR) signaling, and NF-*κ*B/I*κ*B pathway. The Akt signaling pathway has been associated with both viral replication and host survival.^11, 12, 43^

In the present study, we elucidate how NP of IAV interact with and manipulate a host inhibitor of apoptosis API5 during infection, and in the process stimulating E2F1-dependent apoptotic pathway.

We have shown that API5 interacts with NP of IAV and not with other viral proteins such as M1 and NS1, suggesting specificity of interaction with NP (Figure 1a and Supplementary Figure S1). Importantly, the interaction is conserved in H1N1 and H3N2 subtypes, underlining its relevance in IAV pathogenicity (Figures 1a and b). It is worth noting that API5 and NP interact primarily in the nucleus (Figures 2b and d) and incidentally the nuclear localization of API5 is known to be instrumental for its control of E2F1-dependent activation of proapoptotic gene *APAF1*.^30, 31^ Thus, we expected nuclear localization of NP and API5 to have a bearing on *APAF1* transcriptional status. Indeed, NP induced *APAF1* transcription, which was further facilitated by API5 depletion, whereas it was remarkably dampened by API5 overexpression (Figures 5b and c). Importantly, silencing of API5 expression led to E2F1 enrichment on *APAF1* promoter in infected cells (Figure 6). Hence, we propose that interaction of API5 with NP of IAV sequesters it, rendering it unable to oppose E2F1-dependent transactivation of proapoptotic genes. Not surprisingly, proteins encoded by other viruses such as EBNA3C protein encoded by Epstein barr virus and Hepatitis B virus X antigen (HBx) encoded by hepatitis B virus have been previously reported to regulate E2F1-mediated apoptosis in favor of virus.^44, 45^ Previous reports have shown that IAV sequesters host antiapoptotic protein bcl2 through its phosphorylation by activating cellular p38MAPK, facilitating viral-induced apoptosis.^46^

IAV has previously been shown to induce the intrinsic apoptotic pathway,^21^ which involves initiator caspase activation mediated by apoptosome, a complex consisting of cytochrome *c*, APAF1, and dATP, which in turn binds and activates caspases.^47^ NP of IAV has been recently shown to promote the release of cytochrome *c* into cytoplasm through its modulation of clusterin–Bax association.^21^ Previously, it has been reported that M1 protein favors APAF1 expression through its interaction with and sequestration of Hsp70, a known inhibitor of APAF1.^19^ In the present study, we elucidate an emerging role of NP in regulation of APAF1 levels (Figure 4). Importantly, our data suggest that API5 overexpression antagonizes this function of IAV by abrogating E2F1 recruitment to the *APAF1* promoter (ChIP assays, Figure 6), thus preventing its transactivation. There is documented evidence which indicates that caspase 3 activation during the onset of apoptosis is a crucial event for efficient influenza virus propagation.^36^ In agreement with this report, we show that IAV infection and NP expression induce activation of caspases 9 and 3 (Figure 4). API5 knockdown facilitated activation of caspases in IAV microenvironment, highlighting the negative role of API5 in IAV-induced apoptosis (Figure 5). Essentially, API5 downregulates APAF1 and prevents activation of caspases, thus impeding apoptosome formation under infection conditions. Further, IAV employs NP to interact with and sequester API5 along with transcriptionally suppressing it, thus ensuring uninterrupted activation of the apoptotic pathway. Hence, taking all our current observations in view, it appears that different proteins encoded by IAV, such as NP and M1, utilize multiple mechanisms to target strategic host proteins involved in regulation of the cellular apoptotic network for instance Hsp70, clusterin, Bax, and now API5.

Effect of the NP–API5 interaction on IAV-dependent APAF1 expression and caspase cleavage prompted us to check apoptosis induction in NP expressing as well as IAV-infected cells. In agreement with previously reported role of NP in IAV-induced apoptosis, 7AAD, and annexin V staining results indicated higher number of apoptotic cells upon NP overexpression (Figure 7). Interestingly, ectopic expression of API5 either alone or with NP led to a significant reduction in the number of cells undergoing apoptosis, reinforcing the notion that API5 opposes the apoptosis-inducing function of NP. Apoptosis is critical for viral replication and dissemination,^48^ which is also the case with influenza virus replication as overexpression of antiapoptotic factor API5 led to a decrease in viral transcription, which was concurrent with decrease in viral protein levels (Figure 8). Consistently, API5 depletion increased viral polymerase activity and viral titers, further confirming that expression of antiapoptotic protein API5 is detrimental for viral replication and propagation (Figure 8).

Recently, API5 has been shown to play a role in cell cycle progression and cell proliferation by regulating E2F1-dependent transactivation of genes involved in cell cycle progression.^49^ Manipulation of cell cycle and induction of apoptosis are two common strategies used by many viruses to regulate their infection cycles. Although DNA viruses promote cell cycle progression and prevent apoptosis, RNA viruses for example HIV, coronavirus IBV, and influenza virus arrest cell cycle at an early stage of infection.^50, 51, 52^ These reports, together with role of API5 in cell proliferation, raise a compelling possibility that regulation of API5 by NP in IAV-infected cells, apart from its role in apoptosis, may also impact E2F1-mediated cell cycle control. Although it is not the focal point of the present study, it shall be interesting to evaluate the role of API5 in IAV-mediated cell cycle arrest and its repercussions on viral replication.

Conclusively, our findings define a novel regulatory axis active in IAV-induced host cell apoptosis, whereby IAV instructs viral NP to suppress apoptosis inhibitor protein API5, thus ensuring facilitation of E2F1-dependent apoptosis and hence maintenance of viral replication and spread. Our study also amply demonstrate the significance of viral NP in regulating the host cell response, particularly apoptosis. Further, our study warrants quest for more host proteins critical for viral life cycle, the elucidation of which can provide useful insights into designing novel antiviral interventional strategies. Also, novel therapeutic strategies can also be designed to disrupt the NP–API5 interaction, or by disrupting the API5 pathway of apoptosis, as host cells are less likely to undergo mutational changes.

## Materials and Methods

### Cells and cell lines and viruses

HEK293, MDCK, and A549 cell lines were obtained from American Type Culture Collection (ATCC, Manassas, VA, USA) and maintained as per the supplier's instructions (DMEM 10% FCS). A/*Puerto Rico*/8/34 (PR8) and A/*Aichi*/2/1968 (X31) influenza virus strains were used at an MOI of 1 unless specified otherwise for 1 h at 37°C. After 1 h absorption, cells were washed with PBS and were supplemented with DMEM 1% bovine serum albumin (BSA) medium containing 1 *μ*g/ml of tosylsulfonyl phenylalanyl chloromethyl ketone (TPCK)-treated trypsin. Mock infection is carried out with allantoic fluid of uninfected eggs.

### Plasmid constructs, antibodies, siRNA

The NP gene of H5N1 A/Hatay/2004 isolate was cloned into pCDNA 3.1-His plasmid to be used as bait vector for co-IP studies. Full-length human API5 gene cloned in HA and Flag-tagged pLPC plasmid was provided by Nicholas J Dyson.^31^ Anti-NP antibody was obtained from Abcam (Cambridge, MA, USA). Anti-API5, anti-APAF1, anti-procaspase 3, anti-cleaved caspase 3, anti-procaspase 9, anti-cleaved caspase 9, anti-myc, and anti-His antibodies were purchased from Santa Cruz Biotechnology, Inc (Santa Cruz, CA, USA). Anti-Flag, anti-GAPDH, anti-API5, and anti-*β-*actin antibody were purchased from Sigma-Aldrich (St. Louis, MO, USA). Anti-PARP antibody was purchased from Cell Signaling Technology (Danvers, MA, USA). Pool of gene-specific siRNAs against API5 was purchased from Santa Cruz Technologies (Santa Cruz, CA, USA).

### Yeast two hybrid

Lex A-based screening system (Hybrid Hunter, version F), comprised of *Saccharomyces cerevisiae* strain L40 [*MATa his3*Δ*200 trp1-901 leu2-3112 ade2 LYS2::(*4*lex*Aop-*HIS3)URA3*::(8*lex*Aop-*lac*Z) *GAL4]*, pHybLexA/Zeo–B42 and pYesTrp2 as binding domain and activation domain vectors, respectively, and human lung cDNA library cloned in pYesTrp2 was purchased from Invitrogen. Screening was performed as per manufacturer's protocol. The bait plasmid was constructed by cloning IAV NP coding sequence in frame with the LexA DNA-binding domain in pHybLexA/Zeo. PHybLexA/Zeo-NP was co-transformed with cDNA library in L40 and co-transformants were selected for the activation of two reporter genes, *HIS3* and *LacZ*. Strength of the interaction in selected co-transformants were assessed by their ability to grow on His^−^ Trp^−^ and Zeo^+^ YC media supplemented with 5 mM AT (3-amino-1,2,3-trizole, competitive inhibitor of HIS3) and for positivity of filter *β-*galctosidase activity assay. Plasmids were isolated from positive co-transformants and shuttled into *E. Coli* DH5*α* and sequenced. The sequence obtained was analyzed by BLAST to identify their insert. L40 co-transformed with pHybLexA/Zeo-Fos and pYesTrp2-Jun was used as the positive control and L40 co-transformed with pHybLexA/Zeo and pYesTrp2 was used as the negative control for the library screening.^32^

### Western blot analysis

Cells were treated with lysis buffer (50 mM Tris, pH 7.5, 150 mM NaCl, 1 mM EDTA, 0.1% Triton X-100) supplemented with complete protease and phosphatase inhibitor mixture (Roche, Basel, Switzerland) and the lysates thus obtained were subjected to SDS-polyacrylamide gel electrophoresis.

### Co-immunoprecipitation

A549 cells were infected with either mock (allantoic fluid of uninfected eggs), PR8, and X31 virus at an MOI of 1. Cells were collected in the above mentioned lysis buffer, and cell lysates were incubated with the primary antibody overnight followed by 90 min incubation with protein A and G Dynabeads purchased from Invitrogen (Grand Island, NY, USA). The beads were washed three times with chilled PBS, resuspended in Lamelli buffer, boiled for 10 min, and spun down. Supernatants were subjected to western blotting.

### Immunofluorescence microscopy

A549 cells were transfected with either pcDNA3.1-myc/His (control transfection) or pEGFP-NP plasmids using lipofectamine 2000 and following manufacturer protocol (Invitrogen, NY, USA). Cells were fixed at 16 h post transfection with 2% paraformaldehyde in PBS for 20 min at room temperature, followed by permeabilization with 0.4% Triton X-100 for 15 min at room temperature. Cells were then blocked with PBS containing 5% BSA. Immunostaining was performed using rabbit anti-API5 antibodies. Unbound antibodies were washed away with PBS and incubated with mouse anti-rabbit Alexa 594-conjugated antibody purchased from Invitrogen. The nucleus was stained with DAPI. Slides were observed under × 60 magnification of A1R (Nikon, Tokyo, Japan). A549 cells infected with either mock (allantoic fluid of an uninfected egg) or PR8 influenza virus at an MOI of 1 were fixed at 5 and 16 h p.i. in PBS with 2% paraformaldehyde for 20 min at room temperature, permeabilized with 0.4% Triton X-100 in PBS for 15 min at room temperature, and blocked with PBS containing 5% BSA. Immunostaining was performed using mouse anti-NP and rabbit anti-API5 antibodies. Unbound antibodies were washed away with PBS and incubated with goat anti-mouse Alexa 488 and mouse anti-rabbit Alexa594-conjugated antibodies purchased from Invitrogen. The nucleus was stained with DAPI. Slides were observed under × 60 magnification of A1R (Nikon, Tokyo, Japan). The images were captured and analyzed using Nikon AIR Elements software.

### Quantification of *NP*, *APAF1*, and *API5* mRNA by real-time qRT-PCR

Total RNA from cells was extracted using the RNeasy Mini Kit from Qiagen (Valencia, CA, USA), and 2 *μ*g of RNA was reverse transcribed using the ThermoScript RT-PCR System (Invitrogen, CA, USA) in a volume of 20 *μ*l after DNase I treatment. Resulting cDNA was diluted 1 :  10 and 2.5 *μ*l was used in a SYBR Green from SA Biosciences (Valencia, CA, USA) based real-time PCR reaction in a volume of 25 *μ*l using a Mx3000 real-time PCR instrument of Stratagene (Santa Clara, CA, USA). Primers used for real-time RT-PCR are API5 primers forward AGG CAG TAC CCC TCT TCT CTA, API5 reverse CCG CCA ACA ATT TCA ATA CCT CC. NP gene primers (for mRNA), forward CTC GTC GCT TAT GAC AAA GAA G and reverse AGA TCA TCA TGT GAG TCA GAC. APAF1 gene primers, forward CCT CTC ATT TGC TGA TGT CG and reverse TCA CTG CAG ATT TTC ACC AGA; housekeeping gene, *β*-actin with primers, forward ACC AAC TGG GAC GAC ATG GAG AAA, and reverse TAG CAC AGC CTG GAT AGC AAC GTA; GAPDH primers, forward TCA CTG CCA CCC AGA AGA CTG, and reverse GGA TGA CCT TGC CCA CAG C, were used to normalize the Ct values obtained in the real-time PCR reactions, which were then used to calculate fold changes compared with the uninfected sample using the ΔΔCt method.

### Virus infections and API5 silencing

A549 cells plated at a density of 10^6^ per well in a six-well plate were transfected with 80 nM non-targeting (NT) siRNA, 80 nM gene-specific siRNA targeted against API5, pLPC-Flag plasmid (control transfection), and pLPC-Flag-API5 plasmid. At 24 h post transfection cells were infected with A/PR/8/34 at an MOI of 1, and harvested 24 h later. The whole cell lysates were analyzed by western blotting with respective antibodies.

### ChIP–qPCR assay

Chromatin immunoprecipitation (ChIP) assay was carried out as described earlier.^53^ Chromatin obtained was purified using the QIAquick PCR purification kit (Qiagen, Venlo, The Netherlands). The eluted genomic DNA was subjected to SYBR green real-time qPCR with the APAF1 ChIP primers, forward GCC CCG ACT TCT TCC GGC TCT TCA and reverse GGA GCT GGC AGC TGA AAG ACT C. The results were expressed as fold enrichment over control (NT siRNA or pLPC-Flag).

### Flow cytometry

Annexin V and 7AAD staining of cells was done using annexin V PE/FITC apoptosis detection kit (BD Pharmingen, San Jose, CA, USA) according to the manufacturers' instructions. Samples were acquired on BD FACS Calibur (20 000 cells per sample; BD Biosciences, NJ, USA) and analyzed using Flowjo version 9.3.3 software (Tree Star Inc, Ashland, OR, USA).

### Plaque assay

A549 cells were treated either with NT siRNA, API5 siRNA, pLPC-Flag (control transfection), or pLPC-Flag-API5. At 24 h post transfections, cells were infected with X31 virus, a reassortant between PR8 and A/Aichi/68(H3N2) virus, at an MOI of 1. Supernatants collected after 24 h were analyzed for virus growth by plaque assay using MDCK cells. X31, being a low pathogenic virus as compared with PR8, forms clean plaques. MDCK cells were seeded in six-well plates (E106 cells per well) and the plates were incubated at 37°C overnight. Cell monolayers in all six-well plates were washed twice with DMEM and the culture supernatant containing virus was added in a volume of 200 *μ*l at different dilutions. Each dilution was plated in duplicates. Plates were incubated with virus for 1 h followed by washing with DMEM with 0.3% BSA. The cells were overlaid with 1.6% Agarose (SeaKem LE, Cambrex, East Rutherford, NJ, USA) in L15 medium (2 × L15, 1M HEPES, 200 mM Glutamine, 50 mg/ml Gentamyin, NaHCO3, and penicillin streptomycin) with 1 mg/ml TPCK-treated trypsin (Sigma-Aldrich). The plates were incubated for 2–3 days, agarose was removed, cells were fixed with 70% ethanol for 5 min, and stained with crystal violet stain for 30 min. Cells were washed with distilled water, dried, and plaques were counted.

### Cell viability assay

For transfection studies, A549 cells were seeded at 10 000 cells per well in a 96-well dish. After adherence, they were treated with either NT or actinin-4-specific siRNA for 24 h. For infection studies, A549 cells were seeded at 0.8 × 10^6^ cells per well in a six-well plate, after adherence cells were infected with PR8 and X31 at an MOI of 1. Following this, 200 *μ*g/*μ*l of MTT solution/well was added and incubated at 37°C for 30 min to allow for the formation of formazan. The medium was removed, and 200 *μ*l DMSO was added to each well to dissolve the formazan. Absorbance was measured on an ELISA plate reader with a test wavelength of 570 nm and a reference wavelength of 630 nm to obtain sample signal (*A*_570_–*A*_630_). DMSO was used as a reference.

### Luciferase reporter assay

Full-length genomic segments of PB2, PB1, PA, and NP derived from PR8 were cloned in pCDNA3.1. A549 lung epithelial cells were pretreated with NT siRNA and API5 siRNA. Components of influenza polymerase PB2, PB1, PA, and NP were transfected along with luciferase reporter plasmid, which contains noncoding sequences from the NS1 segment of IAV and the luciferase gene driven by pol I, 24 h post transfection of API5 siRNA.^54^

### Statistical analysis

Data are expressed as mean±S.E. Means were compared by one-factor analysis of variance followed by Fisher protected least significant difference to assess specific group differences. Differences were considered significant at *P*<0.05.

## Abbreviations

IAV: Influenza A virus
NP: nucleoprotein
API5: Apoptosis inhibitor 5
MOI: multiplicity of infection
APAF1: Apoptotic protease activating factor 1
