# Supplementary material for: Nucleoprotein of influenza A virus negatively impacts antiapoptotic protein API5 to enhance E2F1-dependent apoptosis and virus replication
Source: Cell Death Dis. 2015 Dec 17;6(12):e2018–. doi: 10.1038/cddis.2015.360 (PMC4720893; doi:10.1038/cddis.2015.360)
Supplement: Supplementary Information [file cddis2015360x1.docx]

**Supplementary Fig. S1**


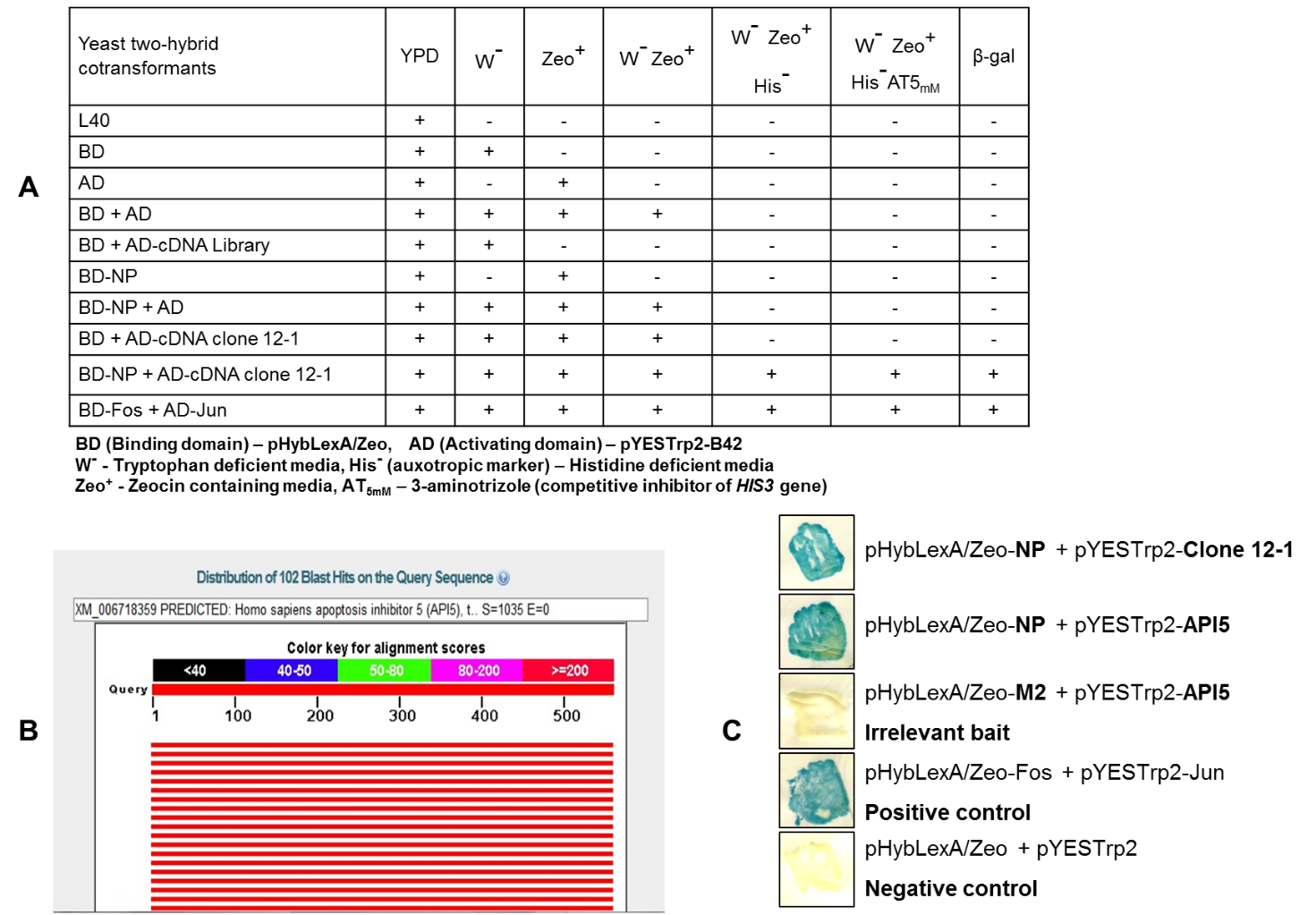


**Fig. S1.** (A) A schematic representation of the yeast-two hybrid screening of lung cDNA library using NP as the bait protein, wherein all columns present conditions under which the screen was carried out. Positive (+) sign represents growth of L40 yeast under given conditions whereas, negative (-) sign represents no growth of L40 yeast, except last column where positive (+) sign represents positive β-gal assay. (B) The putative interacting cDNA clones were subjected to DNA sequencing followed by BLAST analysis to identify their inserts. BLAST results showed that API5 has 100% homology to the sequenced cDNA clone. (C) The full length API5 (pYESTrp2-B42-**API5**) was also checked for Histidine prototrophy against NP (pHybLexA/Zeo-**NP**) as the bait protein, where previously known interactors Fos (pHybLexA/Zeo-**Fos**) and Jun (pYESTrp2-B42-**Jun**) were used as positive controls and empty pHybLexA/Zeo and pYESTrp2-B42 were used as negative controls. In addition, IAV M2 (pHybLexA/Zeo –M2) was used as irrelevant bait against API5 (pYESTrp2-B42-**API5**). BD (binding domain) - pHybLexA/Zeo; AD (activating domain) - pYESTrp2-B42; W^-^ - Tryptophan deficient media; His- (auxotropic marker) - Histidine deficient media; Zeo+ - Zeocin containing media; AT 5mM – 3-aminotriazole (competitive inhibitor for the product of HIS3 gene).
